# ‘A matter of commonsense’: the Coventry poliomyelitis epidemic 1957 and the British public

**DOI:** 10.1080/13619462.2016.1247701

**Published:** 2017-07-03

**Authors:** Gareth Millward

**Affiliations:** ^a^ Faculty of Public Health and Policy, Centre for History in Public Health, London School of Hygiene & Tropical Medicine, London, UK

**Keywords:** Vaccination, public health, medicine, policy, media

## Abstract

In 1956, the British Ministry of Health instituted a vaccination programme against poliomyelitis, but run into myriad supply and administrative issues. When Coventry experienced an epidemic in 1957, it came to symbolise these problems. Throughout, it was claimed that the government lacked ‘common sense’. This article explores how and why ‘common sense’ was used as a rhetorical weapon in the debates over policy at the local and national level. While those claiming ‘common sense’ were often at odds with medical and administrative authorities, the arguments were often informed by deeply held beliefs about vaccination and disease.

In the summer of 1957, Coventry became the subject of national attention when an outbreak of poliomyelitis^1^ struck the city. A series of supply issues had led to a shortage of the new polio vaccine, and many who had registered their children for immunisation were yet to receive their course of injections. Worse, the Ministry of Health refused to divert vaccine from non-epidemic areas to help deal with the crisis, or even to import extra supplies from abroad. Over July, August and September, the press made a series of accusations about government incompetence at the local and national levels, claiming that this was an avoidable tragedy. Many of the criticisms would feel familiar to observers across the post-war period in Britain. The Ministry was, apparently, too bureaucratic, putting procedure above people’s lives; the medical authorities were not quick enough to arrive at concrete solutions to various problems as they arose; more than that, the experts were crippled by a lack of ‘commonsense’.^2^

This article examines how these issues manifested through the debate over polio vaccine supply in the city of Coventry. It focuses on two main questions. First, why did the Ministry of Health refuse to redirect vaccine to Coventry and other epidemic areas to help manage the outbreak? Second, in the face of a nationwide shortage, why did the national government refuse either to seek foreign supplies or to accept offers of help from abroad? To explain this, government papers and press articles are examined at the national and local level, and placed into the wider context of public health at this time.

The debate about Coventry’s epidemic was part of a larger national and even international network of politicians, medical professionals, press and administrators. Its history draws attention to the fact that British public health in the post-war era was not solely a national concern and that there were many actors claiming to represent different publics within this framework. Each drew on different forms of epistemic authority, while also claiming to be working in the interests of ‘the public’ in general terms. In the press, at least, it was clear that the most acceptable solutions to the crisis would have to adhere to the laws of common sense. What is most striking, however, is not whether cultural understandings of disease accorded with the latest scientific consensus or bureaucratic practice. Rather, it is the use of common sense as a rhetorical device, particularly in public discourse. Who used common sense? When did they feel able to employ it? What can this tell us about public attitudes to disease?

## ‘Common’ sense

Anthropologist Kate Crehan notes that ‘common sense’ is, ironically, a common sense concept that few academics have tried to define. Versions of the phrase exist in multiple European languages. In the Anglophonic world, it has been seen as generally positive; yet in Italy, the idea of ‘senso comune’ is a more neutral description of ‘beliefs and opinions supposedly shared by the masses of the population’, including both wisdom and superstition.^3^ This idea of unsaid, generally accepted beliefs pervades through much ethnographic research, too. Anthropologists attempt to delve into the belief systems of communities to get beyond what people literally say to understand what they mean. Here, grasping the ‘common sense’ of a culture distanced by language, place and/or time offers the possibility of richer analyses of various social phenomena.^4^ Historians have made similar attempts to dig deeper into their source materials, trying to discover the unwritten by reading ‘along the archival grain’. There is much to be learned from what was ‘unwritten’ ‘because it could go without saying and “everyone” knew it’, ‘because it could not yet be articulated’ and ‘because it could not be said’.^5^ There is also a wealth of historical research that seeks to show that many parts of society that are believed to be ‘natural’ are very much dependent on historical and cultural context. These historians seek to ‘undermine the authority of what passes for “common sense” in the particular society in which they live and write’.^6^

This article does not attempt to reconstruct Britain’s common sense in 1957. Instead, it focuses on the rhetorical uses of the concept in political discourse. This follows on from Sophia Rosenfeld’s work on common sense as a key weapon in the battles over emerging democratic institutions extending back to at least the Glorious Revolution in the Anglophonic world.^7^ This was most notably expressed in Thomas Paine’s 1776 pamphlet *Common Sense*, but many actors have sought to draw on common sense’s epistemic power.^8^ Any argument that could be tied to a universal knowledge, innate to right-thinking persons, would require a great burden of proof to be overturned.^9^ Indeed, Rosenfeld argues that this is what made the concept central to democratic legitimacy. It validated political decisions made on behalf of the people by rooting them in collective knowledge. Crucially, it also acted as a bulwark against other forms of epistemic authority which may have been a threat to liberal property-owning democracy: namely theology and superstition on the one hand, and abstract philosophy and technocracy on the other.^10^

Yet expertise and common sense have to coexist for the establishment and maintenance of expert-informed policy. On some level, the people have to trust that experts can identify and solve problems in the common interest. Similarly, expertise has to prove its ability to do so, and communicate this effectively to the public.^11^ In the 1950s, this was broadly the case, as post-war Britain had embraced technocratic solutions to long-standing problems. Mike Savage’s work with the Mass Observation archive shows that the middle classes began to see technical expertise as a marker of class identity.^12^ In the realm of public health, ‘evidence’ became used in ‘policy’ increasingly from 1945 onwards. The social medicine research of academics such as Jerry Morris and the epidemiological statistical work of Richard Doll and Austin Bradford Hill revealed (among many other phenomena) the benefits of exercise and the dangers of smoking.^13^ Moreover, there were concrete public health victories that authorities could point to. The national vaccination campaign against diphtheria from 1940 onwards had made a significant impact on morbidity and mortality.^14^ The well-established local authority public health system had also done much to reduce the burden of disease over the course of the interwar period.^15^ To be sure, there was opposition to the technocrat or the specialist as ‘ignorant’ of the wider factors that were required to make political decisions; but, as Michael Edgerton argues, the idea of scientific, technocratic governance was not completely rejected.^16^ Science and common sense were therefore not opposed; it was possible to argue that the use of science and evidence to solve a problem was, in itself, common sense.

When discussing political arguments, however, there is one fundamental and crucial irony. Common sense could only be employed as a political tool when the consensus of universally accepted knowledge broke down. Its users sought to represent the interests of a particular constituency (be they an imagined majority or minority) while simultaneously appealing to the universal wisdom of the entire population. Common sense has often been linked with ‘the public’ and the ‘public good’,^17^ another concept that is supposedly universal but has repeatedly been shown to be fractured.^18^ As debates in the sociology of science have also highlighted, science also does not have a monopoly on truth, nor can its systems of knowledge production always keep pace with ‘the speed of politics’.^19^ Extensive print media, radio and the newly created Independent Television allowed various forms of knowledge to circulate quickly.^20^ In the debate over Coventry’s 1957 epidemic, multiple publics could be invoked in multiple media and, as will be shown, multiple ‘common’ senses. The question is, who was wielding common sense as a weapon, how did they do it, and who did they claim to represent?

This analysis uses internal files from the Ministry of Health and the Medical Research Council (MRC) to investigate the government’s approach. Coventry History Centre also holds the records of the City Council minutes and the Medical Officer of Health’s reports. From the media, a mix of local and national press is considered. The primary national title is that of *The Times*, often referred to as Britain’s ‘paper of record’. It tended to report from the perspective of the British establishment in London. Also included are the *Guardian* (a left-leaning broadsheet), the *Daily Mirror* (a left-leaning populist tabloid) and the *Daily Express* (a right-leaning middle-class tabloid). In the medical press, the British Medical Journal (BMJ) was published weekly and included correspondence from doctors as well as editorials on government policy. At the local level in Coventry, there were two newspapers. The *Coventry Evening Telegraph* (hereafter, *Telegraph*) was published Monday–Saturday, and released in the late afternoon, meaning it often reported on news and events that may have been missed by the national dailies. The *Coventry Standard*, meanwhile, was a weekly paper that went on sale every Friday. It tended to contain more stories about local events and people than its daily competitor, though it did run editorials on the polio epidemic with regard to Coventry’s position within the national picture.

## Salk vaccine and British supply

The reason that Coventry became a rhetorical battle ground in 1957 was bound up in recent medical and technical developments in the treatment and prevention of poliomyelitis. The disease was a relatively new phenomenon, with the first epidemics occurring in Sweden and the United States in the late nineteenth and early twentieth centuries. The first major epidemic in Britain occurred in 1947.^21^ From that point until the 1960s, summers were punctuated with a ‘polio season’, resulting in thousands of cases per year.^22^ Hays uses the term ‘virgin soil infection’ to describe its impact. For while the disease itself is thought to be as old as humanity, it was not until the late 1800s that it became visible, identifiable and hit communities in epidemic fashion.^23^ Polio is transmitted through the gut, and in the majority of cases does not present with symptoms. However, for a minority, it can cause muscle weakness and, in severe cases, paralysis. Although the death rate was higher for adults than children, its ability to confer lifelong weakness or paralysis in children provoked dread in parents. Imagery of children on polio wards inside iron lungs caused similar anxiety. Indeed, the disease was commonly referred to as infantile paralysis.^24^ Polio also caused disquiet among medical professionals because it had become a growing threat despite—perhaps even because of—the ‘successes’ of public health measures and biomedicine over the previous 100 years.^25^ Further, it appeared to affect rich and poor alike and, being viral rather than bacterial, was unaffected by the new ‘magic bullet’ antibiotics of the 1940s and 1950s.^26^

Hopes of eradication (or at least effective control) were placed upon immunisation technology. In 1955, the University of Michigan announced in front of the world’s media that an anti-polio vaccine developed by Jonas Salk had been shown to be safe and effective in a massive trial on American children.^27^ There were three types of poliovirus, and so strains of polio that could immunise against all three were chosen. A strain called ‘Mahoney’ was used against Type-I, ‘MEF-1’ for Type-II and ‘Saukett’ Type-III. The viruses were inactivated using formaldehyde, ensuring that the immune system could respond to the material and develop immunity to poliovirus but that there was no chance of actually infecting the patient.^28^ A full course involved three doses. Within hours, it was licensed for use in the United States. Denmark developed its own form of the vaccine using a similar technique,^29^ while British health authorities also began investigating the possibility of using immunisation as a way of combatting polio. It had to be used as part of a mass vaccination schedule, since immunity took time to develop. While the injected vaccine would take around six weeks to confer protection, ‘natural’ polio could enter through the digestive system and either result in infection or pass through excrement to infect other people. In time, an oral vaccine developed by Albert Sabin would be crucial as it provided immunity in a much shorter space of time and is now used by the World Health Organization as an effective tool for managing epidemics.^30^ However, it was still under development and testing in 1957, and so was not a factor in Coventry.^31^

The polio vaccine came under scrutiny almost as quickly as it had been hailed as a medical marvel. In 1955, a faulty batch of Salk vaccine was released from Cutter Laboratories. The virus had not been inactivated, meaning that children were directly injected with live and potent poliovirus. Confined to the United States, some 400,000 doses were administered, leading to 260 cases and ten deaths.^32^ The Cutter Incident, as it became known, caused trepidation for a number of governments across the developed world.^33^ The British authorities were particularly keen to ensure that standards of safety and efficacy were guarded. Initial trials of the vaccine in the country were halted in the summer of 1955, and the MRC sent investigators to America to determine what had gone wrong. In the British parliament, MPs sought assurances that the safety of British citizens would be safeguarded.^34^ More importantly, authorities were unwilling to risk tainting the reputation of vaccination in general, which had been a key tool in reducing rates of diphtheria, tuberculosis and smallpox in particular.^35^

Regardless of Cutter, the British medical authorities were convinced that it was possible to make a more potent and safer version of an inactivated polio vaccine. In particular, they felt that the ‘Mahoney’ strain of polio that had been isolated and inactivated in Salk’s vaccine was not as efficient as the Type-II and Type-III strains being used. It was also failure to inactivate Mahoney that had caused the Cutter Incident. Research suggested that even though the risk of complications from the American vaccine was very slight, another Type-I strain may reduce these risks even further. British manufacturers began synthesising a new product based on the Brunden poliovirus strain, which, testing had shown, met these criteria.^36^ Additionally, the MRC and Ministry of Health were convinced that British safety testing procedures were more robust than those across the Atlantic. Partially this was built on national pride at the historical quality of British medicine, and partly as a result of Cutter. Medical authorities had been similarly cautious over the introduction of Bacillus Calmette–Guérin (BCG, the antituberculosis vaccine) and diphtheria immunisation, preferring instead to rely on tried-and-tested public health systems or disinfection, quarantine and hospital care. The fact that these were foreign technologies had caused trepidation, especially in the case of BCG.^37^ Testing procedures also took longer in Britain and were more thorough, suggesting a higher standard of potency and efficiency.^38^ The Ministry was therefore reluctant to use a vaccine that was potentially ‘unsafe’ when compared to the British version—even though the risks of either had been shown to be low, and even though two of the three strains in the vaccine were identical. Concurrently, officials believed that it was important for British economic interests to have a strong, alternative vaccine to the Americans’. The government worried that if Britain was slow to exploit it, this lucrative new market could potentially leave it frozen out later in the century.^39^ There is little evidence in the MRC papers that this was born out of any specific ‘anti-American’ sentiment; though it is hard not to see elements of insecurity stemming from Britain’s changing role as a world power. The Eden and later Macmillan governments were certainly concerned about British diplomatic and financial interests on the world stage; and the pharmaceutical industry was certainly one area in which Britain could remain influential.^40^ On this basis, the Ministry refused to allow local authorities to use the American product as part of their general vaccination programmes, justified by ‘medical evidence’ from the MRC.^41^ This claim would be contested during the 1957 epidemic.

After reviewing the evidence on Cutter and concluding that the incident was a result of manufacturing error rather than a fundamental flaw in inactivated vaccine technology, the MRC trials restarted in late 1955.^42^ They suggested that the British vaccine was safe and effective. The Eden government committed itself to a vaccination programme involving children aged under ten in January 1956 in what became known as the Turton announcement (after then Health Minister Robin Turton).^43^ This meant that the refusal to use Salk created some significant technical problems.^44^ First, British companies did not have the manpower or laboratory space to produce enough of the vaccine. Only two companies—Glaxo and Burroughs Wellcome—had been licensed to make the vaccine, and the latter had faced significant delays. Only Glaxo was producing and releasing the vaccine for public consumption during the Coventry epidemic.^45^ Registration rates for the newly announced programme were relatively low (around 29 per cent of those eligible),^46^ and the British version only required two doses; but even so, manufacturers found it difficult to establish a reliable supply chain. It became clear that Britain and British pharmaceutical companies could not produce the vaccine quickly enough to reach everyone before the polio season began that summer.^47^ Not only were batches of the vaccine difficult to produce, they had to go through a testing procedure that could take around three months, so any delays in manufacture had knock-on effects. There was also limited testing capacity, meaning that the whole system acted like a ‘pipeline’. One batch had to be manufactured, added to the testing queue and then cleared before work could start on the next.^48^ In an attempt to overcome the shortages, Glaxo was asked by the Ministry of Health to increase its production by 50 per cent. It agreed.^49^ Yet, the company was mindful about the immense costs involved in competing for specialists from an emerging (and rather small) pool of talent, and the ‘possible future redundancy of staff and capital assets’ once the first generation of children was vaccinated.^50^ There may have been millions of children and young adults to vaccinate in the next few years, but, once this backlog was cleared, demand would effectively fall to the annual birth rate. Besides, even if Glaxo did set up a new laboratory, it would take between six and twelve months to build, plus another six before the first batch was produced, tested and ready for use.^51^

The British government was thus in a precarious position, partly of its own making. Even allowing for the arguments over increased safety and efficiency, the programme of vaccinating the under tens left them very little margin for error. If a batch was delayed or faulty, there would not be enough supply to vaccinate all those registered before the polio season began in the summer.^52^ Unfortunately for the Ministry, this happened in February 1957 when a batch of the Glaxo vaccine changed colour while in transit, suggesting that a foreign body had contaminated the vials. Immediately, production was halted while the medical authorities investigated.^53^ *The Times*’s reaction was unfavourable.

This frustrating series of official announcements can give rise to nothing but concern, not only among the parents of the [c. 1.7 million] registered for vaccination last year and still awaiting the oft-promised vaccine but also among family doctors and medical officers of health. […] What should be made abundantly clear is that these delays are in no way the fault of the MRC or [Glaxo]. […] What seems to have happened is that the Minister took a gamble and announced the probable date on which the vaccine might be ready, knowing full well that no one could give any guarantee.^54^

The BMJ was similarly scathing. It accused the government of using polio vaccine as a public relations exercise, and, initially at least, failing to inform the medical press about the specifics of the vaccine. Offended by the ‘American’ style conference that had initially proclaimed the programme, it quipped that ‘the dream-like stage … proved to be more fitting than seemed possible at the time.’^55^

It is into this context, then, that the 1957 epidemic in Coventry was born. The national press was already agitated by what appeared to be mismanagement by the Ministry of Health. Thousands of children who ought to have been vaccinated by the time of the 1957 polio season had yet to complete their course of injections. And, through it all, supplies of the vaccine arrived slowly, with no reserve and no hope of ramping up production in the near future. Having promised that 300,000–500,000 children would be fully vaccinated with two doses by June 1957, just 200,000 had been by the beginning of March, many with only a single injection.^56^

## Why Coventry?

Coventry had found itself victim to these supply issues, with only 6,000 of its 11,000 registered children receiving the vaccine.^57^ This became politically sensitive when an epidemic ran through the city in the summer of 1957. Coventry was in Warwickshire, around 25 kilometres east of Birmingham and 140 kilometres north-west of London.^58^ Over the twentieth century, it had become a centre for car and motor cycle manufacturing. During the 1950s, a massive rebuilding project had been required due to heavy damage sustained during devastating German bombing raids in 1940. The city’s population was expanding, both as a result of declining mortality rates and migration from Ireland and other areas to work in the motor industry.^59^ Despite the national Conservative government, Coventry had a predominantly Labour council, and all three of the city’s Members of Parliament (MPs) represented Labour. Two of these, Maurice Edelman and Richard Crossman, were nationally recognised. Crossman was a political heavyweight, and would go on to be the key architect of social security policy in the Wilson governments of the 1960s.^60^ Edelman, meanwhile, was an author who was adept at using the media. He had written plays for the newly formed Independent Television Association, and would be the main campaigner in Parliament for Coventry during the epidemic.^61^ The ‘crisis’ in Coventry played out over the course of just a few months, with most of the news stories being printed in the first weeks of August. While the epidemic itself was newsworthy, Edelman’s machinations undoubtedly focused national attention. As Drakeford and Butler have argued, the existence of a problem is not enough to make it a political scandal. Some group needs to articulate why the general population should care, and it needs to capture the public mood.^62^ Coventry did not have the worst rates of polio per capita in the country, but its plight was framed as the consequence of chronic government mismanagement.^63^ In Edelman, the City had an articulate national spokesman to make this case, and, whether by design or by coincidence, he created a scandal that could not be ignored.

The press in Coventry reported an upsurge in polio cases in mid-July. Medical Officer of Health, Dr. Thomas Morris Clayton, made announcements and undertook interviews to help disseminate public health advice.^64^ Concern had been raised as the number of cases up to that point implied that the summer of 1957 would see more poliomyelitis than the previous worst year for the disease in the city, 1953. Clayton wrote to the Ministry of Health asking for more supplies of polio vaccine to help build greater immunity in the population. On Friday, 2 August, Clayton told the City that this request had been denied.^65^ At this point, Maurice Edelman, the MP for Coventry North, took up the matter in Westminster. He announced that he would meet with the Ministry and make another request. After a telephone call on Saturday morning, he declared that the government had changed its mind and would be providing more vaccine for Coventry. His success was reported in the Saturday evening edition of the *Telegraph*.^66^ Some local and national news followed suit on Monday morning.^67^

These reports came as a surprise to the Ministry of Health, where officials were convinced that Edelman had been told nothing of the sort. They spent most of Saturday informing the news agency Extel that there had been no change of policy, and convinced the BBC and ITV not to run with the story in their evening news bulletins. They also issued denials to the newspapers that had run the story.^68^ Whether Edelman was lying, twisting the facts or had misinterpreted the Ministry is open to interpretation. The government had announced that a new batch of vaccine was on its way, and that Coventry would be getting its share.^69^ In a sense, Coventry was getting more vaccine; just nothing over and above what it would have received in normal circumstances. On the other hand, when a civil servant spoke to the *Telegraph* to clarify the story, he quoted the paper: ‘We are not surprised. We never believed the Edelman story anyway; he only believes what he wants to believe’. This certainly was an opportunity for Edelman to launch a campaign of local significance, as well as allowing a prominent opposition MP to attack the government of the day. His constituency was also the most heavily hit by the disease.^70^ Regardless, the news of extra vaccine had been given a warm reception by the press, and when this was found to be false it provoked confusion and resentment.^71^ Edelman’s announcement drew attention to the problems within the vaccination programme.^72^ It was reported as if the government was sticking to its policies in the face of ‘commonsense’.^73^

Common sense was a recurring trope in the coverage of the epidemic, as it has been in political discourse for centuries.^74^ Here, it was claimed by a number of different constituencies. Medical authorities appealed for calmer heads and recognition of the need for compromise at times of shortage. The local press and politicians, meanwhile, argued that technocratic and bureaucratic rigidity was overriding the wishes and rights of the city’s population. Throughout, common sense would be invoked in different ways by different authorities claiming to represent different constituencies (or ‘publics’). To demonstrate this in the context of the Coventry epidemic, two distinct, but entwined, issues are worth considering: the refusal of the Ministry of Health to redirect vaccine supplies to Coventry; and the refusal to accept vaccine from foreign suppliers when it was offered.

## Redirection

The common sense response from local politicians and press was that populations ought to receive vaccination in times of epidemic. This was seen as a defence against further infection, and a way of containing the spread of infectious disease. Such tactics had been used, alongside quarantine and sanitation methods, to combat outbreaks of smallpox. It was also well established by law and precedent that local public health authorities, with the support of the national government, would undertake such operations. Thus, was it not ‘common sense’ that supplies of the vaccine should be funnelled to Coventry and other epidemic-affected regions?

The Ministry argued that it could not redirect extra supplies to Coventry. It had no reserves, since the vaccine was distributed as soon as it was approved.^75^ Each local authority received a proportion of each batch relative to the number of registered people in the area. Further, it was unwilling to divert vaccine away from places which had not yet developed an epidemic for fear of the political consequences should one occur. Every locality, therefore, had already received its ‘fair share’.^76^ Indeed, the government went so far as to say Coventry had received even more than others on the basis of its high registration rate. Forty per cent of those eligible had registered, compared to the national average of 29. By comparison, Warwickshire’s rate was 43 per cent, neighbouring Staffordshire 24, and Birmingham 30.^77^

Even if both sides of this argument accepted the general principle of vaccination, other common sense attitudes led to different political conclusions. For the national authorities, it was logical and self-evident that compromises had to be made in times of scarcity. The local medical administrator agreed. Thomas Clayton, Coventry’s Medical Officer of Health, responded to the government’s rejection in the press by stating:

We would obviously like to be in possession of a great deal more vaccine. It is a positive and important means of preventing cases occurring. But we are not in a position of unlimited supplies so we have to apply commonsense.^78^

Those claiming to represent citizens, however, were not impressed. Edelman had a meeting with John Vaughan-Morgan, the acting Minister of Health,^79^ and told the press:

I pleaded that the commonsense of the public would favour emergency measures to deal with an emergency area and that it was necessary to take a slight risk in diverting supplies.^80^

Invoking ‘commonsense’ and ‘the public’ at this point exposes a number of intriguing issues. In terms of the local public, Edelman was on strong ground. Editorials in and letters to the *Telegraph* and *Standard* showed local demand for more vaccine.^81^ At the national level, however, there was room for debate. Perhaps Edelman was correct in assuming that the public in polio-free areas would be willing to suspend their vaccination programme in the short term. The *Daily Mirror* argued that the government’s stance was ‘fair … in normal conditions. But to insist on it now is high-bound officialdom’.^82^ For Vaughan-Morgan, on the other hand, the problem was not the public of today, but an imagined public of the future should the Ministry take an active decision to redirect supplies from an area that later on in the summer could develop an epidemic. ‘The public’ was an important rhetorical tool, one that could shift in place and time depending on the argument being made.^83^

There were sound technical reasons for overruling the common sense of the people of Coventry. The solution to a smallpox epidemic was not necessarily correct for poliomyelitis. Clayton argued that the press had ‘gone too much to town’ on vaccine supplies, overemphasising the short-term benefits.

Every Medical Officer would welcome greater supplies of the vaccine as it was the only practical measure that could be taken to combat polio in present conditions. But this was not going to alter the present situation at all. It might take two or three months to get immunity into a person. It was not for the present epidemic, but as a future preventative measure.^84^

This information was used by both sides of the debate. An editorial in the *Telegraph* still argued that it would be ‘a matter of commonsense’ to divert the vaccine—even if it took six weeks to develop immunity, this would help the city by the end of the polio season.^85^ The Ministry, on the other hand, stressed that the nation’s resources would be better used across the whole population rather than in local circumstances. The *Daily Express* showed sympathy with Coventry, but ultimately concluded ‘there is not enough [vaccine]—and anyway it probably would not help’.^86^

Medical advice also suggested that vaccination was counterproductive in epidemic situations. The Joint Committee on Polio Vaccination (a policy advisory body mainly of officials from the Ministry of Health and the Scottish health authorities) and the World Health Organization both argued that vaccination ought to be suspended in the wake of a serious epidemic.^87^ Medical opinion had only recently begun to shift away from this stance. There was evidence to suggest that immunisations led to an increase risk of paralysis at the site of the injection. The national diphtheria advertising programme had been muted during the summer months as a precaution in the early 1950s as a result, and it was not until 1956 that the MRC advised the government that it would be acceptable to run the polio immunisation programme during the ‘polio season’.^88^ As the Ministry told Edelman in a letter printed in the *Telegraph*:

Anxiety naturally stimulates a local demand for emergency measures. But it would be quite wrong to deal on such a footing with a problem which is unpredictable and affects all parts of the country. […] Vaccination is not generally accepted as being a suitable emergency measure to check and contain local outbreaks.^89^

Clayton had been annoyed that his call for more vaccine at a previous press conference had been interpreted as an endorsement of vaccination as an immediate solution to all the City’s problems.^90^ In this climate, ‘commonsense’, the wisdom of ‘the public’, was not a useful basis for national policy, at least as for the Ministry. Instead, a dispassionate view, taken on the national level and buttressed by the advice of medical bodies, was more important than the demands of local people, justified by populist understandings of vaccination and infectious disease. The Ministry also had a long-term outlook, expressing its desire to maintain the integrity of the vaccination programme as a whole. It could not be sacrificed for local, short-term and acute cries for help. Yet, the government still had to be mindful of the public’s reaction to such policies.

## Refusing importation

It became clear very early into the crisis that the Ministry was not going to redirect supplies away from other areas. After Clayton’s and Edelman’s requests were denied in early August, Coventry’s attention focused elsewhere. One solution was to increase national supply so that each local authority’s slice of the metaphorical pie became larger. Yet the government refused to import from North America, where a number of companies were manufacturing the Salk-type vaccine. Even when it was given direct offers, it turned these away, too.

At three points during the summer, Coventry was presented with opportunities to use foreign-made vaccine. The first was in early August when the Danish paper *Berlingske Tidende* ran a campaign to raise money to buy polio vaccine from the government of Denmark. The country had a history of providing vaccination aid to other nations, and the Coventry story had made some impact in Copenhagen.^91^ However, the appeal was soon dropped. ‘We were told an official demand must come before anything could be done’, said O Mikkelsen, the paper’s London correspondent, ‘and that would not come from England because of the British attitude on importing vaccine’.^92^ Another came from the Pasteur Institute, which proposed to donate a French-made vaccine to Coventry. Edelman had liaised with the French laboratory and presented this to the Ministry of Health, in part due to his role as chair of the Franco-British Parliamentary Relations Committee.^93^ Much like in Britain, French authorities were developing a new type of vaccine that was supposedly safer than the American version.^94^ The Ministry investigated the possibility, but soon concluded that it was ‘unacceptable in a number of ways’.^95^ There was ‘no evidence of satisfactory studies in human volunteers’, no controlled trial, and the vaccine contained penicillin and streptomycin which in the view of the Ministry ‘involves danger to the health of the community’. Although designed to help avoid contamination of vials, no thorough testing had been done on the safety of such agents in vaccines.^96^ Edelman accepted the decision, but argued that more still needed to be done to secure better supplies.^97^ He had succeeded in causing another headache for the Ministry, however. This time it was a diplomatic incident with the French government, unhappy at the apparent snub of French medical science and offer of aid in a time of crisis.^98^

The most instructive case involved American vaccine. Charles Braham, a Coventry businessman with links to the United States, came forward claiming to have an agreement with American firms to import Salk vaccine for use in the city. Clayton refused ‘with grateful thanks’.^99^ He knew the Ministry would never allow it—as it confirmed with a statement that ‘the type of vaccine to be used in this country is a national matter and not one for decision by individual health authorities’.^100^ When the issue of supply was raised, Britain’s refusal to import was regularly cited as part of the problem. In the press and the wider public it did not seem credible to continue the embargo. Salk was considered safe enough for doctors to use on patients if they had received the vaccine as a ‘gift’ from the United States; but was not considered appropriate for the mass vaccination programme. Although designed to allow an element of flexibility for in a minority of cases, these contradictions caused further confusion and agitation.^101^ Again, the appeal was made to the common sense of ‘experience’: for, ‘surely [Salk vaccine] has been proved by experience on a vast scale in the United States to be effective and safe’?^102^ How could Salk not be ‘safe enough’ despite ‘more than 100 million people’ being injected with it with no problems?^103^ ‘Informed opinion is aghast at the rigid attitude of the Ministry’, claimed the *Telegraph*, and pointed to a *Lancet* article that called the Salk ban ‘either unsafe, or unduly harsh, and in any case irrational’.^104^

The public face of the Ministry was accused of being ‘obscurantist’.^105^ In part, this was because there were delays in the internal decision-making process. As late as July 1957, the government believed ‘the best course of action’ was ‘to continue to resist pressure for the purchase of American vaccine, but to step up production in this country’.^106^ It was clear, however, that this position was increasingly untenable. Lord Alec Douglas-Home, the Lord President of the Council,^107^ and Vaughan-Morgan began corresponding with Sir Harold Himsworth, Secretary of the MRC, on the matter.^108^ It had become ‘a matter of political importance’,^109^ and if domestic supplies could not be secured, ‘pressure would revive for the importation of the Salk vaccine from the United States’.^110^ As the summer went on, the government sought direct medical advice from the MRC to justify an import of Salk. The MRC, however, felt unable to do so. This caused a rhetorical conundrum, which was solved through announcements that allowed the government to confirm its future policy whilst not contradicting its previous stance. The MRC advised that the ban on Salk was no longer medically justified; but it stopped short of actively calling for imports to begin.^111^

This allowed the MRC to declare that it felt Salk was safe enough to use on the British people, and also permitted the government to place orders for the vaccine. Popular and medical opinion backed this stance. Salk had been shown since Cutter to be very effective in reducing polio morbidity and mortality, and no major reports of injuries had come since 1955. The MRC and BMJ, however, still stressed that the British vaccine was safer (even though the risks from both were still slight), both because of the strain of virus used and the more stringent testing procedures in the UK.^112^ Thus, the MRC stressed that Salk was an emergency measure; one that could be justified because the use of American product to immunise the people was better than suspending or slowing the national programme. In normal circumstances, the government should be making every effort to ensure British vaccine was available for all, as this was still the safer and more potent option. If the government did decide to import, the MRC issued the following caveats: Salk ‘should be imported only as a temporary measure to bridge the gap between need and adequate supply of British vaccine’; any ‘vaccine purchased by public money should be subjected to safety and potency tests in this country’; and those receiving the vaccine should be advised ‘it is not entirely devoid of risk’.^113^ In theory, this gave British parents a choice about whether they felt it better to have their children immunised quicker with foreign vaccine, or to wait for the home-grown product. The MRC’s position on British vaccine and the superiority of British testing procedures was therefore reaffirmed, while still allowing the government to continue the programme and address public concerns.

In the meantime, the government continued to gather information to make sure that there definitely would not be a shortfall for 1958. It was resigned to the fact it could do little in the current epidemic, but, given the testing procedures that were considered necessary, it had little time to place an order for American vaccine. While it would be difficult, though justifiable, to write off 1957, ‘it would become increasingly difficult to justify our leaving numbers of children un-protected next year’.^114^ Briefing papers from early September showed that 16.5 million extra doses were required, even allowing for increased output from Glaxo and Burroughs Wellcome and no further production problems. Thus, on 11 September, the government announced it would be placing orders for Salk vaccine. At the same time, and despite the problems it had experienced with children under ten, it announced that the vaccination programme would be extended to all children aged under 15 .^115^

Even with the supply issues, highlighted in the press as early as February, the government took many months to finally agree to import Salk. These delays were seen by the *Telegraph* as ‘an extraordinary example of the ‘red tape’ mentality’^116^ and *The Times* agreed that ‘in the absence of a reasoned statement the citizens of Coventry cannot be blamed if they fail to understand why the offer of Salk vaccine made to them [by Braham] should have been rejected by the Ministry’.^117^ Both the *Guardian* and *Daily Mail* showed some sympathy with the government’s predicament, but both concluded that this was a crisis caused by its mishandling of supplies.^118^ The government had already overridden the wishes of the local public by refusing to redirect supplies to Coventry, deferring to the wider national interest. Was it now continuing to defy the common sense of ‘informed opinion’, which the *Telegraph* declared was ‘aghast at the rigid attitude of the Ministry in dealing with Coventry and other places’?^119^ Why did the government not take ‘a more realistic view’?^120^ Were these pronouncements on the safety of Salk simply ‘to cover up [the Ministry’s] earlier error in failing to import it?’^121^ On these points, the criticism was rather contradictory. Despite their calls for more vaccine supplies, *The Times* acknowledged that Salk and other vaccines were not a useful form of epidemic control; rather, they were needed to prevent future outbreaks.^122^ The *Standard*,^123^ *Daily Mail* ^124^ and the City’s Medical Officer of Health^125^ made similar observations. Moreover, when parents were given the opportunity to register for the ‘safer’ British vaccine, most had not taken it. Now, in the wake of an epidemic, opinions had seemingly reversed. As the *Guardian* made clear, ‘it should be remembered that the public, too, [had] blown hot and cold about the virtues of vaccination’.^126^ The BMJ commented on this curious state of affairs, noting that perhaps the medical community had not done enough to inform ‘public opinion’ on the relative merits of different forms of the vaccine, either in the past or in the present crisis.^127^

The British public and medical authorities had indeed blown ‘hot and cold’ on immunisation, and not just in 1957. It appeared that common sense could very quickly be brought into view by circumstances. England in particular had a long history of anti-vaccination sentiment from the nineteenth century, stemming in large part from opposition to compulsion measures in Victorian public health legislation.^128^ Much of this had waned by the post-war era following the end of compulsion. But newer immunisations for diseases more common than smallpox and based on modern biomedical principles proved to be more popular. The national anti-diphtheria campaign, for example, reduced morbidity and mortality from the disease significantly after its introduction in 1940. 12.5 million children were immunised between 1940 and 1956, reducing the case load from around 58,000 in 1940 (with 2,800 deaths) to just 51 cases (and 8 deaths).^129^ Even here, however, the British government had been wary about introducing such measures, believing that experience with smallpox vaccination showed there would be opposition from the public. It was only when they were thoroughly convinced that the technology was safe and—crucially—more effective than existing, time-tested public health measures that immunisations against tuberculosis and diphtheria were introduced. This was despite, in the case of both vaccines, evidence to suggest their effectiveness in other western nations.^130^ Moreover, diphtheria immunisation revealed that parents were yet to fully embrace immunisation as a ‘fact of life’ and routine element of childhood. In the wake vastly reduced rates of diphtheria, the number of children under the age of 12 months immunised against the disease dropped from over 75 per cent in 1949 to just 28 per cent in 1951.^131^ The Ministry of Health had high hopes that newer vaccinations against whooping cough and polio would see greater demand because parents still feared those diseases. But again, polio stands as an example of how parents were hesitant to give a new, untested vaccination to their children. As mentioned previously, Coventry’s registration rate of 40 per cent was relatively high. It was only in the wake of an epidemic—seemingly fuelled by government mishandling—that it became ‘common sense’ to increase the availability of vaccine.

Once again, the government took a long-term view of the vaccination programme. Importing Salk was not going to have a material impact upon the Coventry epidemic. Even if orders had been placed as soon as the crisis began, the insistence on testing meant that it would have been months before it could be used in the city. Similarly, the epidemic appeared to have created a ‘panic’. Was it good policy to respond to every surge in demand? Could the voice of common sense lose its edge when it appeared to be driven by immediate, panicked reactions to an acute crisis rather than the available evidence?^132^ Here, then, debates around common sense were refracted through the lens of the parties hoping to invoke it. For the national press, Coventry became an example of what could happen to any one of the local authorities in the country. Taking a more pragmatic view, national supplies had to be ameliorated, and common sense dictated that these come from North America.^133^ In Coventry, acute demand drove both the call for Salk vaccine in the short term and in the medium term to ensure such a situation did not occur in the near future.

## Conclusion

Alderman S Stringer’s assessment of the 1957 epidemic was apt: ‘polio disease was not a Coventry question—nor merely a question for Great Britain—it was international’.^134^ Citizens were caught and constrained in a number of technical and bureaucratic networks, involving American and British pharmaceutical companies, the Ministry of Health and the City Council. The *Telegraph*’s reflections on the epidemic made a last appeal to ‘commonsense’, perhaps one thing that the local population could still keep within its control. When it had failed to gain significant policy concession in the immediate term with regard to vaccine supply, the City had focused primarily on nuisance removal and cleaning rivers, canals and pools.^135^ In pondering whether more could have been done at both the local and national level, the paper wrote that:

the policy to combat polio must be pursued out of season with the zeal that is felt now. … If all of us as a community would be more scrupulous so as to make many of these tasks unnecessary, how much better would it be. No-one knows what causes a polio epidemic, but in the name of commonsense let us not be open to the suspicion that we may have brought it on ourselves.’^136^

To a certain extent scandals are, as Drakeford and Butler have shown, manufactured.^137^ Coventry suffered 106 cases of the disease, out a population of around a quarter of a million.^138^ More had been reported in 1953 without the same level of media and public scrutiny.^139^ At that time, there were attempts to downplay the condition as mild, with ‘the fear of polio … often worse than the complaint’.^140^ Councillors warned the press and others not to ‘scare the public’.^141^ But 1957 was different. Coventry was in the midst of what could be portrayed as a failure of national, technocratic government. Indeed, that was the basis of the crisis rather than the disease itself. In 1953, there had been no vaccine; in 1957 there was one, but its delivery system had cracked due to poor maintenance. In short, it was possible for Edelman and others to claim their solutions to the government’s mismanagement were ‘commonsense’. They found an outlet for these views. In part, this was because of the strength of feeling over polio and concern over how the vaccination programme was being run. However, as Vaughan-Morgan lamented, ‘unfortunately, also, the peak period of the disease is in the late Summer, and coincides with the “silly season” in the Press’.^142^

Cultural memory of vaccination and public understanding of infectious disease helped to mould common sense. Thus, opinions and policies that drew on these memories could be framed as common sense and used to defend a political position. For the local press and politicians opposed to government policy, common sense offered protection against attitudes that were seen as technocratic, uncaring, ‘rigid’ and ‘unduly harsh’.^143^ The wisdom of the public could go up against medical and expert knowledge to fight for concessions. At the same time, ‘informed opinion’ offered a spin on common sense.^144^ Still rooted in the reasoning and logic of the people, the *Lancet* could criticise government policy,^145^ while the BMJ could argue that, once the public understood some of the key details, their opinions would be swayed.^146^ This exposes a core tension in many of the arguments. Those wanting to wield common sense needed enough expert evidence to avoid the charge of ignorance, while simultaneously appealing to the innate knowledge and reasoning skills of the common man. They needed enough emotion to break down the heartless technocratic approach to disease prevention, but not so much that they appeared to be knee-jerk, panicked reactions.

This article, however, has not been concerned over the correct levels of information or emotion required to produce common sense. Instead, it has focused on the rhetorical use of the concept.^147^ What mattered, therefore, is not whether a particular argument really was common sense, but whether it could be claimed as such. With so many different constituencies and spokespeople claiming to represent them, there could also be a range of common senses. Edelman may have tried to manipulate the debate to his own ends, but he could claim the ground of common sense with a number of his criticisms of government policy. Equally, Vaughan-Morgan and other medical authorities at the local and national level could provide evidence and logic to deny Edelman’s requests. In some areas, the opposition’s arguments effected change—after all, the government eventually began importing more vaccine. Yet the government’s stance on redistribution won out, aided by medical and expert evidence that defied initial common sense conclusions.

Common sense arguments are powerful because they require a high burden of proof to overturn them.^148^ This does not mean they cannot be overturned; nor does it mean that experts and their knowledge are antithetical to common sense. Technocratic or bureaucratic arguments, when put in the correct terms, were an ample defence for the government in the face of opposition and dissent. This may have been due to the relatively high regard with which expertise was treated in the 1950s. Further study is needed on which forms of ‘expertise’ were able to sway common sense in different times and circumstances. But it should be noted that the idea of using a mass vaccination programme, centrally controlled and based on cutting-edge medical technology, was at the heart of this entire crisis. From the sources consulted, nobody seriously challenged the idea of a technocratic solution to the scourge of poliomyelitis in Britain. Common sense was employed as a watchdog, to make sure technocracy remained fair and worked how it was supposed to. There was not a simple battle between the rational and the irrational. Rather, the two were intertwined and inseparable.

## Notes on contributor

***Gareth Millward*** is a research fellow at the Centre for History in Public Health at the London School of Hygiene and Tropical Medicine. He is currently working on a Wellcome Trust funded project entitled Placing the Public in Public Health: Public Health in Britain, 1948–2010. His current research focuses on the post-war vaccination programme in Britain and its relationship with the public.

## Funding

Wellcome Trust Investigator Award, held by Alex Mold. [grant number WT-100586-Z-12-Z].

## Notes

1. Hereafter polio.

2. This is a direct quotation, following the single-word style of the *Coventry Evening Telegraph* (hereafter *CET*) in the 1950s. See: “Help Needed Now.” *CET*, 7 August 1957, 6.

3. Crehan, “Gramsci’s Concept of Common Sense”.

4. Headland et al., *Emics and Etics*; and Jardine, “Etics and Emics”.

5. Stoler, *Along the Archival Grain*, 3.

6. Rosenfeld, *Common Sense*, 2.

7. Rosenfeld, *Common Sense*; Rosenfeld, “Before Democracy”.

8. Paine and Kramnick, *Common Sense*. On the philosophical history of the concept, see also: Ben-Chaim, “Locke’s Ideology of ‘Common Sense’”; Jackson, “Coleridge, ‘Common Sense,’ and the Literature of Self-experiment”; and Lemos, *Common Sense*.

9. Lemos, *Common Sense*, 4–6.

10. Rosenfeld, “Before Democracy”.

11. Carmargo and Grant, “Being Right is Not Enough”.

12. Majima and Savage, “Contesting Affluence: An Introduction”; and Savage, “Affluence and Social Change”.

13. See the edited volume: Berridge, ed., *Making Health Policy*. Also: Smith, “Uses of Epidemiology”; and Centre for History in Public Health, “Epidemiology”.

14. Lewis, “The Prevention of Diphtheria”; The National Archives (hereafter TNA): BN 10/229, Ministry of Health, Immunisation against Diphtheria 1957–1958.

15. Welshman, “Watchdog or Lapdog?”; Gorsky, “Public Health in Interwar Britain”.

16. Edgerton, “Revisiting the Technocratic Moment,” esp. 188–189.

17. Wilson argues that this became a key feature of Georgian politics in Britain. Wilson, *The Sense of the People*, 1.

18. On the plurality of ‘the public’, especially in relation to public health and medicine, see: Sturdy, “Medicine, Health and the Public Sphere,” 16; Cantor “Representing ‘the Public’”; Gilbert, “Producing the Public”; and Newman and Clarke, *Publics, Politics and Power*.

19. Collins and Evans, *Rethinking Expertise*, esp. 7–8.

20. The latter had been introduced with political controversy and claims that it would damage public discourse. See: Freedman, “The Labour Party and the Origins of ITV”.

21. Smallman-Raynor and Cliff, “The Geographical Spread of the 1947 Poliomyelitis Epidemic”.

22. Public Health England and Department of Health, *Immunisation against Infectious Disease*; Oshinsky, *Polio*, esp. 1–12.

23. Hays, *Epidemics and Pandemics*, x, 414.

24. ‘Polio’ had become the more popular nomenclature after the Second World War, but, despite the fact that the disease did affect adults, its association with childhood was part of its threat. See: Paul, *A History of Poliomyelitis*, 8.

25. It has been hypothesised that in less-hygienic societies, children are exposed to the poliovirus at a very young age, when they may still have some passive immunity from the mother’s immune system. After the arrival of better sanitation, especially in densely populated environments, children and adults were not exposed to the virus until a later age, by which point they were at greater risk of developing symptoms. Oshinsky, *Polio*, 22–23, 28–31; Straus and Straus, *Medical Marvels*, 164–167.

26. A cure for polio has never been found. Hays, *Epidemics and Pandemics*, 377, 415–416. See also Rogers, *Dirt and Disease*; Rogers, *Polio Wars*; Gareth Williams, *Paralysed with Fear*; and Offit, *Vaccinated*.

27. The results were published as Francis, *Evaluation of the 1954 Field Trial of Poliomyelitis Vaccine*.

28. Anon, “Assessment of the British Vaccine Against Poliomyelitis”; Salk et al., “Safety Testing of Experimental Poliomyelitis Vaccines”.

29. Engels et al., “Cancer Incidence in Denmark Following Exposure to Poliovirus Vaccine Contaminated With Simian Virus 40”.

30. Kew et al., “Endgame Strategy for Global Polio Eradication”.

31. For more on this and the international networks involved in polio vaccine see Vargha, “Polio Vaccination across the Iron Curtain”; and Williams, *Paralysed with Fear*.

32. Nathanson and Langmuir, “The Cutter Incident”; and Offit, “The Cutter Incident, 50 Years Later”.

33. Axelsson, “The Cutter Incident and the Development of a Swedish Polio Vaccine”; Day, “An American Tragedy”; and Porras and Cabellero, “Vaccines and Vaccination against Smallpox and Poliomyelitis”.

34. “Vaccine trials postponed.” *The Times*, 18 June 1955, 5; *Parliamentary Debates (Commons)*, 540, 2 May 1955, 1327–1328; ibid*.*, 540, 4 May 1955, 141 W; ibid*.*, 542, 27 June 1955, 35-6 W; and ibid*.*, 543, 4 July 1955, 65-6 W.

35. TNA: FD 23/1058, Harold Himsworth to G S Wilson, 23 July 1957; and Anon, “Vaccination Against Poliomyelitis”.

36. TNA: FD 23/1028, MRC Advisory Committee on Safety Tests for Poliomyelitis Vaccine, Minutes, 16 May 1957; Memorandum on the Importance of Gaining Answers to Two Questions Concerning the British Salk-Type Poliomyelitis Vaccine (late 1956), 1–6; and Anon, “Assessment of the British Vaccine Against Poliomyelitis”.

37. Bryder, “We Shall Not Find Salvation”; and Lewis, “The Prevention of Diphtheria”.

38. TNA: FD 23/1028, MRC Advisory Committee on Safety Tests for Poliomyelitis Vaccine, Minutes, 16 May 1957; Memorandum on the Importance of Gaining Answers to Two Questions Concerning the British Salk-Type Poliomyelitis Vaccine (late 1956), 1–6.

39. TNA: FD 23/1058, Copy of extract of Cabinet Conclusions C.C.(57)66th Conclusions of Tuesday 16th September 1957, Item No. 1; Glaxo Ltd. And Polio Vaccine (1957).

40. Ruane and Ellison, “Managing the Americans”.

41. TNA: FD 23/1028, MRC Memorandum—Poliomyelitis Vaccine, Early 1957; and ibid*.*, Telephone Conversation with Mr. E.D.T. Jourdain, Lord President’s Office, 10 September 1957.

42. Anon, “Assessment of the British Vaccine Against Poliomyelitis”.

43. “British poliomyelitis vaccine in use this summer.” *The Times*, 20 January 1956, 8.

44. For a brief discussion of this issue in Britain, the Netherlands and West Germany, see Lindner and Blume, “Vaccine innovation and adoption”.

45. TNA: FD 23/1028, MRC, Advisory Committee on Safety Tests for Poliomyelitis Vaccine, Minutes, 16 May 1957.

46. “Coventry Not Worst Affected District.” *CET*, 7 August 1957, 1.

47. “British Poliomyelitis Vaccine in Use this Summer.” *The Times*, 20 January 1956, 8; and *Parliamentary Debates (Commons)*, 548, 26 January 1956, 56 W. Originally it was felt that vaccinating during the polio season may risk further infection, but experience in other countries convinced the government to allow vaccination during the summer at a local authorities’ discretion. Anon, “Poliomyelitis Vaccination to Continue During the Summer”.

48. TNA: FD 23/1031, Brief for meeting with Scientific Correspondents. History of Batch 10 (for conference held 27 February 1957).

49. TNA: FD 23/1058, Polio Vaccine. Brief for Lord Hallisham for Ministerial Meeting, 30 August 1957; and Cabinet Home Affairs Committee H.A.(57) 17th Meeting, 19 July 1957, 10am.

50. TNA: FD 23/1058, Glaxo Ltd. And Polio Vaccine (1957).

51. ibid.

52. TNA: FD 23/1028, The use of poliomyelitis vaccine in 1957. A memorandum by Professor A. Bradford Hill (late 1956); and ibid*.* A. Bradford Hill to Sir John Charles, 12 October 1956.

53. TNA: FD 23/1031, Brief for meeting with Scientific Correspondents. History of Batch 10 (for conference held 27 February 1957).

54. “Vaccine Issue Held up.” *The Times*, 28 February 1957, 8.

55. Anon, “Polio Fantasies”. It referenced the international press conference that greeted Salk’s announcement of the successful field trials in the United States. The Ministry of Health had tried a similar exercise with the Turton announcement in January 1956. Anon, “Another Ministry Blunder”.

56. Anon, “Polio Fantasies”.

57. “Polio: Coventry Vaccine Plea Fails.” *CET*, 2 August 1957, 12. This was broadly in line with the national average.

58. Although part of the historic county of Warwickshire, since the reorganisation of local government in the 1970s Coventry is in West Midlands.

59. Ewart, “Coventry Irish”; and Tom Pockock, “The Unlucky City.” *Daily Express*, 8 August 1957, 4. According to the census, the city’s population was 258,211 in 1951. In 1957, the mid-year estimate was 277,300. Coventry History Centre: T. Morrison Clayton, *The Health Services of Coventry in 1957*, 8.

60. Crossman, *The Diaries of a Cabinet Minister. Vol.3*; Howard, “Crossman, Richard Howard Stafford (1907–1974)”; and The Labour Party, *National Superannuation*.

61. Edelman’s papers are available from the Modern Records Centre, University of Warwick, Coventry.

62. Drakeford and Butler, “Everyday Tragedies”.

63. Maidstone was the worst affected local authority, and Lincoln was also noted for the severity of its epidemic. See: “Coventry not Worst-affected District.” *CET*, 7 August 1957, 1.

64. “17 New Polio Cases Reported in Coventry.’ *CET*, 22 July 1957, 3; and “Five New Cases of Polio in Coventry.” *CET*, 31 July 1957, 1.

65. “Polio: Coventry Vaccine Plea Fails.” *CET*, 2 August 1957, 1.

66. “Coventry to Get More Polio Vaccine.” *CET*, 3 August 1957, 1.

67. The government was aware of stories in the *Daily Express*, *Daily Telegraph* and *Birmingham Evening Dispatch.* TNA: MH 55/2460, Polio Vaccine—Coventry, internal memorandum, 6 August 1957, 4.

68. TNA: MH 55/2460, Polio Vaccine—Coventry, internal memorandum, 6 August 1957, 1–2.

69. TNA: MH 55/2460, Polio vaccine for Coventry, Press handout given over telephone by Mr. Heald this afternoon, 5 August 1957.

70. Coventry History Centre: T Morrison Clayton, Medical Officer of Health Report 1957, 44.

71. See esp. “Help this City.” *Daily Mirror*, 7 August 1957, 2.

72. “Polio vaccine: M.P.’s Protest to Ministry.” *CET*, 5 August 1957, 1; “Coventry Plea for Extra Vaccine.” *The Times*, 6 August 1957, 6; “Comment.” *Daily Mail*, 10 August 1957, 1; “Vaccine and Epidemic.” *Guardian*, 12 August 1957, 4; and *passim*.

73. “Help Needed Now.” *CET*, 7 August 1957, 6.

74. Ben-Chaim, “Locke’s Ideology of ‘Common Sense’”; Jackson, “Coleridge, ‘Common Sense,’ and the Literature of Self-experiment”; Rosenfeld, “Before Democracy”.

75. Anon, “Poliomyelitis Vaccine”.

76. “Polio Vaccine: M.P.’s Protest to Ministry.’ *CET*, 5 August 1957, 1; and “Coventry Must Wait, MINISTER tells M.P.” *CET*, 7 August 1957, 3.

77. “Coventry not Worst-affected District.” *CET*, 7 August 1957, 1.

78. “Polio: Coventry Vaccine Plea Fails.” *CET*, 2 August 1957, 1.

79. Vaughan-Morgan was an under-secretary in the Ministry, but was effectively the minister due to the ill health of Dennis Vosper. He would be replaced in September by Derek Walker-Smith.

80. “Coventry Must Wait, MINISTER Tells M.P.” *CET*, 7 August 1957, 3.

81. “Help Needed Now.” *CET*, 6 August 1957, 6; “Three New Cases in Coventry.” *CET*, 8 August 1957, 1; “Refused Again.” *CET*, 9 August 1957, 12; Frank G. Smith, “Go all Out for Salk.” Letter in *CET*, 10 August 1957, 6; and “Minister Refuses Extra Vaccine.” *Coventry Standard*, 9 August 1957, 5.

82. “Help this City.” *Daily Mirror*, 7 August 1957, 2.

83. Newman and Clarke discuss various constructions of ‘the public’ in relation to political debate in the introduction to Newman and Clarke, *Publics, Politics and Power*.

84. “Polio: Lord Mayor Holds Conference.” *CET*, 7 August 1957, 1, 12. Clayton gave a similar statement to *The Times*: “Civic Talks on Poliomyelitis.” *The Times*, 8 August 1957, 2.

85. “Help Needed Now.” *CET*, 7 August 1957, 6.

86. “No Extra Vaccine for Polio Towns.” *Daily Express*, 8 August 1957, 5.

87. “It’s Bad to Inoculate for Polio at Present.” *Coventry Standard*, 9 August 1957, 5; and Anon, “Poliomyelitis Vaccine”. It was felt that vaccination may make patients more susceptible to ‘natural’ polio if exposed. Sabin’s oral polio vaccine would be used in the 1960s for epidemic prevention, but was not yet available.

88. On diphtheria see: TNA: INF 12/238, Meeting to discuss the Diphtheria Immunisation Publicity Campaign, 15 October 1952; and Bradford Hill and Knowelden, “Inoculation and Poliomyelitis”. On polio, see Anon, “Poliomyelitis Vaccination to Continue During the Summer”.

89. “Why Coventry Cannot have Extra Vaccine.” *CET*, 8 August 1957, 9. The letter also appears in TNA: MH 55/2460.

90. TNA: MH 55/2460, Clayton to J. P. Dodds, Ministry of Health, 9 August 1957.

91. Denmark’s vaccine industry had survived the German occupation, and Scandinavian countries were involved in a massive United Nations vaccination campaign against tuberculosis directly after the Second World War. See: Altink, “Fight TB with BCG”.

92. “Danish 5000 ‘Shots’ Campaign Fails.” *CET*, 8 August 1957, 8.

93. TNA: MH 55/2460, Edelman to Vaughan-Morgan, 28 August 1957; TNA: FD 23/1058, MRC Memorandum, 28 August 1957; “Vaccine Offer: Ministry Men Going to Paris.” *CET*, 28 August 1957, 2; “French anti-polio Vaccine Offer.” ibid*.*, 3; ‘Pasteur Institute’s Vaccine Offer.’ *The Times*, 29 August 1957, 5; ‘French Offer of Polio Vaccine.’ *Guardian*, 29 August 1957, 1; “France Makes Polio Offer.” *Daily Mirror*, 29 August 1957, 7; and Anon, “Medical News”.

94. Porras and Cabellero, “Vaccines and Vaccination against Smallpox and Poliomyelitis”.

95. “French Vaccine Offer Rejected.” *CET*, 11 September 1957, 1.

96. “Use of French Product ‘not justifiable’.” *The Times*, 12 September 1957, 10.

97. “French Vaccine Offer Rejected.” *CET*, 11 September 1957, 1.

98. TNA: MH 55/2460, Derek Walker-Smith, Minister of Health to A Maroselli, French Secretary of State for Health, 26 September 1957.

99. “Review of Polio Situation.” *The Guardian*, 9 August 1957, 1. See also ‘Polio City Says ‘No’.’ *Daily Mirror*, 9 August 1957, 3.

100. “Health Ministry Officers in City for Polio Talks.” *CET*, 9 August 1957, 1.

101. This decision was taken because it was easier to police, the number of doses being imported were minimal, and the government did not want to be seen to be too officious. TNA: FD 23/1058, Vaughan-Morgan memorandum to Cabinet Home Affairs Committee, (H.A.57(87)), 17 July 1957; “Polio Attacks Doctors.” *Daily Express,* 6 August 1957, 1; and Anon, “Poliomyelitis Vaccine”.

102. “Help Needed Now.” *CET*, 7 August 1957, 6.

103. “Polio Attacks Doctors.” *Daily Express*, 6 August 1957, 1.

104. “Refused again.” *CET*, 9 August 1957, 12; and “Health Ministry Officers in City for Polio Talks.” ibid*.*, 1. Margaret Agerholm, the author of the *Lancet* article repeated many of these claims in “Poliomyelitis Vaccine.” Letter to *The Times*, 31 August 1957, 11.

105. “For Lack of Vaccine.” *The Times*, 9 August 1957, 7.

106. TNA: FD 23/1058, Note of H.A.(57)79, Cabinet Home Affairs Committee, 3 July 1957.

107. A position within the British cabinet, usually without a specific portfolio. Home had been the minister reporting to cabinet about the anti-poliomyelitis vaccination programme.

108. TNA: FD 23/1058, See: Himsworth to Home, 18 July 1957; Cabinet Home Affairs Committee H.A.(57) 17th meeting, 19 July 1957, 10am, and *passim*.

109. TNA: FD 23/1058, Lord Home to Vaughan-Morgan, 15 July 1957.

110. TNA: FD 23/1058, H.A.(57) 16th meeting minutes, 8 July 1957, Cabinet Home Affairs Committee.

111. TNA: FD 23/1058, Himsworth to Wilson, 23 July 1957.

112. Anon, “Assessment of the British Vaccine Against Poliomyelitis”; Anon, “American Polio Vaccine”; Anon, “British Poliomyelitis Vaccine” (1956); Anon, “British Poliomyelitis Vaccine” (1957); and *passim*.

113. TNA: FD 23/1058, Vaccination against Poliomyelitis, Considerations relating to the possible use of American Salk vaccine in this country, Himsworth to Lord Home, 25 July 1957, 5.

114. ibid*.*

115. TNA: FD 23/1058, Appendix. Poliomyelitis vaccination. Government announce new plans, 11 September 1957; “Salk Vaccine from America ‘Immediately’.” *CET*, 11 September 1957, 1; and “Poliomyelitis Vaccine Coming from U.S.” *The Times*, 12 September 1957, 10.

116. “Refused Again.” *CET*, 9 August 1957, 12.

117. “For Lack of Vaccine.” *The Times*, 9 August 1957, 7.

118. “Vaccine and Epidemic.” *Guardian*, 12 August 1957, 4; “Comment.” *Daily Mail*, 10 August 1957, 1.

119. “Refused again.” *CET*, 9 August 1957, 12.

120. “Sensible decision.” *CET*, 12 September 1957, 10.

121. “The Big Cover Up.” *Daily Express*, 10 August 1957, 5.

122. “For Lack of Vaccine.” *The Times*, 9 August 1957, 7.

123. “Polio Outbreak in Coventry Causes Widespread Alarm.” *Coventry Standard*, 9 August 1957, 5.

124. “The Polio Problem.” *Daily Mail*, 10 August 1957, 1.

125. “Polio: Lord Mayor Holds Conference,” *CET*, 7 August 1957, 1, 12.

126. “Comment.” *Daily Mail*, 10 August 1957, 1; “Vaccine and Epidemic.” *Guardian*, 12 August 1957, 4.

127. Anon, “American Polio Vaccine”.

128. Durbach, “Bodily Matters”; Porter and Porter, “The Politics of Prevention”; Williamson, *The Vaccination Controversy*.

129. TNA: BN 10/229, Ministry of Health, Immunisation against Diphtheria 1957–58.

130. Bryder, “We Shall Not Find Salvation”; and Lewis, “The Prevention of Diphtheria”.

131. TNA: BN 10/229, HK Ainsworth [Ministry of Health] to all Local Authorities, England, 23 May 1952.

132. On the history of an ‘emotional’ public and its supposed negative and positive effects on behaviour, see Cantor, “Representing ‘the Public’”.

133. Canada had also been using Salk, directed by the government and bolstered with locally sourced vaccine. See Oshinsky, *Polio*, 219–220.

134. “Council Parties Agree on Polio and Slums.” *CET*, 4 September 1957, 3.

135. “Publicity and Polio.” *CET*, 16 August 1957, 12.

136. “Avoiding a Next Time.” *CET*, 4 September 1957, 8.

137. Drakeford and Butler, “Everyday Tragedies”.

138. By immediate contrast, 399 cases of tuberculosis were reported that year. Coventry History Centre: T. Morrison Clayton, *The Health Services of Coventry in 1957*, 19, 44.

139. ibid*.*

140. Edgar Letts, “Coventry’s Outbreak of Poliomyelitis Examined.” *Coventry Standard*, 11 September 1953, 6. Other articles in the *Standard* and *CET* reported the outbreak and noted that it was the worst in Coventry’s history, but the volume of pieces is much lower than the 1957 epidemic.

141. “55 Children had Polio: Nearly All will Recover.” *Coventry Standard*, 23 October 1953, 7.

142. TNA: MH 55/2460, Vaughan-Morgan to the Prime Minister, 28 August 1957.

143. “Help Needed Now.” *CET*, 7 August 1957, 6; “Health Ministry Officers in City for Polio Talks.” ibid*.*, 9 August 1957, 1.

144. “Refused Again.” *CET*, 9 August 1957, 12.

145. “Health Ministry Officers in City for Polio Talks.” *CET*, 9 August 1957, 1.

146. Anon, “American Polio Vaccine”.

147. Rosenfeld, *Common Sense*; Rosenfeld, “Before Democracy”.

148. Lemos, *Common Sense*.
